# Beyond large-effect loci: large-scale GWAS reveals a mixed large-effect and polygenic architecture for age at maturity of Atlantic salmon

**DOI:** 10.1186/s12711-020-0529-8

**Published:** 2020-02-12

**Authors:** Marion Sinclair-Waters, Jørgen Ødegård, Sven Arild Korsvoll, Thomas Moen, Sigbjørn Lien, Craig R. Primmer, Nicola J. Barson

**Affiliations:** 1 Organismal and Evolutionary Biology Research Programme, University of Helsinki, Helsinki, Finland; 2 Institute of Biotechnology, University of Helsinki, Helsinki, Finland; 3 AquaGen, Trondheim, Norway; 4 Department of Animal and Aquacultural Sciences, Faculty of Biosciences, Norwegian University of Life Sciences, Ås, Norway; 5 Centre for Integrative Genetics, Department of Animal and Aquacultural Sciences, Faculty of Biosciences, Norwegian University of Life Sciences, Ås, Norway

## Abstract

**Background:**

Understanding genetic architecture is essential for determining how traits will change in response to evolutionary processes such as selection, genetic drift and/or gene flow. In Atlantic salmon, age at maturity is an important life history trait that affects factors such as survival, reproductive success, and growth. Furthermore, age at maturity can seriously impact aquaculture production. Therefore, characterizing the genetic architecture that underlies variation in age at maturity is of key interest.

**Results:**

Here, we refine our understanding of the genetic architecture for age at maturity of male Atlantic salmon using a genome-wide association study of 11,166 males from a single aquaculture strain, using imputed genotypes at 512,397 single nucleotide polymorphisms (SNPs). All individuals were genotyped with a 50K SNP array and imputed to higher density using parents genotyped with a 930K SNP array and pedigree information. We found significant association signals on 28 of 29 chromosomes (*P*-values: 8.7 × 10^−133^–9.8 × 10^−8^), including two very strong signals spanning the *six6* and *vgll3* gene regions on chromosomes 9 and 25, respectively. Furthermore, we identified 116 independent signals that tagged 120 candidate genes with varying effect sizes. Five of the candidate genes found here were previously associated with age at maturity in other vertebrates, including humans.

**Discussion:**

These results reveal a mixed architecture of large-effect loci and a polygenic component that consists of multiple smaller-effect loci, suggesting a more complex genetic architecture of Atlantic salmon age at maturity than previously thought. This more complex architecture will have implications for selection on this key trait in aquaculture and for management of wild salmon populations.

## Background

Characterizing genetic architecture is instrumental for understanding how quantitative traits will change in response to evolutionary processes such as selection, genetic drift, and/or gene flow. Reciprocally, knowledge of genetic architecture can help to elucidate how evolutionary processes lead to particular genetic architectures of quantitative traits, i.e. a few major effect loci (oligogenic) [1, 2], many small-effect loci (polygenic) [3, 4], or genome-wide effects (omnigenic) [5, 6]. Empirical examples that demonstrate a variety of genetic architectures for quantitative traits are accumulating for species such as humans [7] and domesticated animals [8]; however, there remains a limited number of characterized genetic architectures for complex traits in wild species. One factor that hampers characterization of genetic architectures, particularly in wild species, is limited sample size. As sample size decreases, the minimum effect size that can be detected increases [9]. This limitation likely led to a bias in the reported distribution of the genetic architecture for quantitative traits in wild species, for which either very large effect loci are reported [1], or a highly polygenic architecture is concluded due to a lack of any significant association being detected [10].

Aquaculture-reared Atlantic salmon offer an opportunity to overcome sample size limitations. Tens of thousands of individuals are routinely reared in a common environment, from fertilization to maturation. As Atlantic salmon are recently domesticated (just 10 to 15 generations ago) [11], the genetic basis of many quantitative traits in domesticated Atlantic salmon is likely shared with that of wild populations. Recently, the genetic basis of sexual maturation has been extensively studied in Atlantic salmon due to its importance in both aquaculture and the wild [1, 12–19]. In the wild, maturation is a critical point in an individual’s life history, since it affects fitness-related traits such as growth, survival, and reproductive success [20]. Large variability in age at maturity is observed in Atlantic salmon, with some individuals returning to their natal rivers to spawn after just 1 year at sea and others spending multiple years at sea before returning (e.g. [21]). Individuals that delay maturation and spend more years feeding at sea can have a much larger body size and, thus, higher potential fecundity [20], compared to individuals that spend only 1 year at sea. However, individuals that spend multiple years at sea increase their likelihood of mortality prior to maturation and spawning compared to individuals that spend less time at sea and return to spawn at a smaller size [22]. Variability in age and size at maturity is thought to have evolved in order to maximize fitness in highly variable river and ocean environments [23]. This variability also aids in population stability in the face of environmental change and stochasticity via the portfolio effect—a phenomenon where biological diversity within a species reduces population size fluctuations [24].

Although beneficial in the wild, variation in age at maturation can be problematic in Atlantic salmon aquaculture. Early maturation, as soon as 1 year post-smoltification (referred to as *grilsing*), causes significant losses in revenue [25] because flesh quality degrades during the maturation process [26], which can also negatively impact fish health [27]. For this reason, characterizing the genetic basis of maturation in Atlantic salmon has been of particular interest for aquaculture producers. In addition, knowledge of the genetic architecture of maturation in aquaculture strains will provide insights into the genetic basis of maturation in wild Atlantic salmon and potentially other fish species.

Quantitative trait loci (QTL) mapping and genome-wide association studies (GWAS) have both been used to identify genetic variation associated with age at maturity in Atlantic salmon [1, 12–17, 19]. Using 220K SNP genotypes on 1404 individuals from 57 European populations and genome resequencing data on 32 individuals, Barson et al. [1] identified a large-effect locus on chromosome 25, *vgll3*, which explained 39% of the phenotypic variation in sea age at maturity for wild European Atlantic salmon. The *vgll3* gene is an adiposity regulator and is also associated with age at maturity in humans [28, 29]. Another region on chromosome 9 was also strongly associated with maturation, however, it did not remain significant after population stratification correction, suggesting that this region may be associated with a correlated trait (e.g. body size) that is affected by a common environmental factor (e.g. river catchment area) [1, 30]. This region contains a transcription factor of the hypothalamus-pituitary–gonadal axis (*six6*), which is also associated with height and age at maturity in humans [28, 29] and involved in regulating puberty in cattle [31]. In North American Atlantic salmon, late maturation alleles of *vgll3* are observed in higher proportions in late-maturing females than in early-maturing females, suggesting that *vgll3* may also be associated with age at maturity in North American salmon [18]. QTL studies on aquaculture fish did not identify any QTL on chromosome 25, but did find two QTL (chromosomes 10 and 21) for early maturation in males [12]. However, GWAS on aquaculture salmon that followed found a significant association between the *vgll3* region and maturation in some [13, 15] but not all [17, 19] aquaculture strains. In addition, markers located on almost all other chromosomes have shown an association with maturation timing in some studies but not in others [13, 16, 17, 19]. These discrepancies among studies could be due to false positives or, alternatively, false negatives in studies that are under-powered to detect smaller effect loci due to low sample sizes, or they may reflect population differences in genetic architecture. Therefore, in spite of substantial improvement in our understanding of the genetic architecture of maturation timing in Atlantic salmon over the last decade, uncertainties remain regarding the details of its genetic architecture.

Here, we conducted a large-scale examination of 11,166 males from a single year-class that were genotyped with a 50K SNP array. We imputed up to a higher density using parents that were genotyped using a 930K SNP array, combined with pedigree information. Using a GWAS, we aimed at further resolving the genetic architecture of maturation timing in male Atlantic salmon and identifying potential candidate genes to provide new insight into the mechanisms involved in determining age at maturity in Atlantic salmon.

## Methods

### Sample collection and phenotyping

Fish were sampled from the Norwegian AquaGen Atlantic salmon breeding line. This population is the outcome of a breeding program that began in the 1970’s and stems from crosses of founder individuals that originated from 41 wild Norwegian rivers [11]. Pedigree information was available from the breeding programme. In total, 11,379 individuals were collected: 11,166 male offspring from the 2015 year-class and an additional 213 parents from the 2012 parental year-class. Association testing was not conducted on females because the prevalence of early maturation in females in this population is very low. The sample set of male offspring from the 2015 year-class consisted of 578 full-sibling families, with a mean size of 20 (range: 1 to 64), and 213 half-sibling families with a mean size of 106 (range 1 to 206). Maturation phenotype was scored using visual assessment for presence of maturation characteristics (developed kype and darkened colouration) for all males from the 2015 year-class as a binary trait: either grilse (early-maturing) or non-grilse. Scoring occurred 27 to 30 months post-fertilization, during the winter months (December-March), when visible differences between mature and immature fish are strong, and prior to any selection of individuals for optimal growth. Early-maturing individuals were preferentially selected for genotyping to increase effective sample size for downstream GWAS analyses. As a result, the prevalence of early maturation in our study sample is higher than in the population as a whole.

### Genotyping and quality control filtering

The 2015 year-class individuals, consisting of 2104 grilse and 9062 non-grilse, were genotyped using a custom 50K SNP Affymetrix array developed for Atlantic salmon. The SNPs on the 50K array are a subset of those included on the 930K XHD *Ssal* array (dbSNP accession numbers ss1867919552–ss1868858426) that is described in Barson et al. [1]. The 930K SNP array was used for genotyping 184 parents from the 2012 year-class. The remaining 29 parents were genotyped using the 50K SNP array.

The 930 K XHD *Ssal* array was filtered to 646,528 SNPs based on genotyping quality (categories PolyHighResolution and NoMinorHom), minor allele frequency (MAF) higher than 0.001, and correct allele segregation in family material of 840 individuals sampled from the AquaGen strain. All SNPs used in downstream analyses were positioned based on the Atlantic salmon reference genome (assembly ICSASG_v2) [32].

### Genotype phasing and imputation

Pre-phasing of the reference panel of 184 parents that were genotyped with the 930K SNP array was performed using Beagle 4 [33, 34]. Individuals genotyped with the 50K array were imputed to the pre-phased reference panel (646,528 SNPs) using SHAPEIT v2 [35] and the duoHMM method, which incorporates pedigree information to improve phasing accuracy [36]. We used a window size of 5 Mb for defining haplotypes and incorporated pedigree information, which consisted of 889 parent–offspring duos and 10,248 mother-father-offspring trios from 40 families. Prior to imputation and phasing, we removed 773 SNPs from the 50K array that were not on the 930K reference panel. Following phasing and imputation, 134,131 SNPs with a MAF lower than 0.01 were removed using PLINK 1.9 [37], leaving 512,397 SNPs for downstream analyses.

To assess the accuracy of imputation, we masked genotypes in the 184 parents that had been genotyped with the 930 K array. Masked genotypes consisted of 930K array SNPs that were not on the 50K array. In addition to the parents genotyped with the 930K array that served as a reference panel, individuals with masked genotypes were included as extra individuals in the imputation process described above. We then compared the imputed genotypes to the actual genotypes for the 184 parents and calculated the mean proportion of discordance between imputed and actual genotypes at each site using the *diff*-*site*-*discordance* function in *vcftools* [38].

### Genome-wide association testing

We tested for associations of SNP genotypes with age at maturity of males from the 2015 year-class using the linear mixed model method BOLT-LMM [39], which accounts for population structure and relatedness based on the following model:

$${\varvec{\upgamma}} = {\mathbf{x}}_{{{\mathbf{test}}}} \beta_{\text{test}} + {\mathbf{X}}_{{{\mathbf{GRM}}}} {\varvec{\upbeta}}_{{{\mathbf{GRM}}}} + {\mathbf{e}},$$

where ${\varvec{\upgamma}}$ is the vector of phenotypes (0/1 for non-grilse/grilse); ${\mathbf{x}}_{{{\mathbf{test}}}}$ is the vector of genotype codes (0/1/2) for the SNP being tested, which was modelled as a fixed effect with $\beta_{\text{test}}$ as regression coefficient; ${\mathbf{X}}_{{{\mathbf{GRM}}}} {\varvec{\upbeta}}_{{{\mathbf{GRM}}}}$ is the genetic effect modelled as a random effect, where ${\mathbf{X}}_{{{\mathbf{GRM}}}}$ is a matrix of genotypes and ${\varvec{\upbeta}}_{{{\mathbf{GRM}}}}$ is a vector of SNP effects; and ${\mathbf{e}}$ is the vector of residual errors, modelled as random effects. BOLT-LMM implements a non-infinitesimal model that does not assume equal effect sizes and can, therefore, better accommodate SNPs of large effect, while still effectively modelling smaller genome-wide effects. BOLT-LMM uses two prior distributions of effect sizes (${\varvec{\upbeta}}_{{{\mathbf{GRM}}}}$) to accommodate both large-effect SNPs and small-effect SNPs [39]. Since it is known that age at maturity of salmon does not have an infinitesimal architecture [1], we chose a model with increased power to detect signals when the architecture is non-infinitesimal and consists of loci with differing effect sizes. The genetic effect, ${\mathbf{X}}_{{{\mathbf{GRM}}}} {\varvec{\upbeta}}_{{{\mathbf{GRM}}}}$, accounts for the confounding effects of relatedness and family structure [39]. ${\mathbf{X}}_{{{\mathbf{GRM}}}}$ was computed using BOLT-LMM for each SNP tested, using a subset of SNPs that fulfilled the following criteria: from the 50K SNP array and not imputed; showing no evidence of high linkage disequilibrium (LD) to reduce confounding effects of redundant genotypic information; and not located on the same chromosome as the test SNP to avoid proximal contamination [40]. SNPs in high LD (*r*^2^ > 0.8) were identified with PLINK 1.9’s *indep*-*pairwise* function [37] using a 1-Mb window size and a 10-kb step size. The resulting association statistics were calibrated using the LD score regression intercept, as implemented in BOLT-LMM. We estimated LD scores for all SNPs using LDSC [41]. The genome-wide significance level of 9.8 × 10^−8^ for *P*-values was determined using the strict Bonferroni correction (α = 0.05/# of association tests). The linear regression beta coefficients and corresponding standard errors from BOLT-LMM were transformed to odds ratios using LMOR [42].

### Identifying candidate genes

Multiple SNPs within a region can show a significant association due to LD around a causal SNP, but they are not all independently associated with the trait. One approach to account for this LD is to select only the top-associated SNP within the region, but this can fail to identify instances where true secondary signals exist within the region. To overcome this challenge, we performed conditional and joint analyses with the *cojo*-*slct* function [43] implemented in GCTA v1.91.6 [44]. The *cojo*-*slct* function converts marginal effect sizes from a single-SNP association test to joint effect sizes by incorporating information of covariance among SNPs based on the LD structure. Joint effect sizes and *P*-values were calculated conditional on other SNPs, using a stepwise procedure, beginning with the top-associated SNP and iterating over all remaining SNPs [43]. *P*-values and beta values obtained from the BOLT-LMM association test were analyzed with *cojo*-*slct* to identify independently associated SNPs. We considered a SNP to be independently associated with the maturation phenotype if the conditioned *P* value was less than 9.8 × 10^−8^. Linkage disequilibrium was calculated using the genotyping data (512,397 SNPs) of all 11,166 male offspring.

Any SNP that was significantly associated with age at maturity in the conditional and joint analyses was assigned a candidate gene. A SNP that was located within a gene region was assigned to that gene, otherwise the nearest gene (within 50 kb upstream or downstream) was assigned. If a SNP was located within two overlapping genes, both genes were considered candidates. We used the function *closest* implemented in BEDTools (v2.26.0) [45] to assign candidate genes to SNPs. Genes and gene locations were based on the current Atlantic salmon genome assembly (ICSASG_v2) [32]. RefSeq annotations for Atlantic salmon genes (available at: https://www.ncbi.nlm.nih.gov/gene/) were used to determine the overlap between candidate genes for age at menarche in humans [28, 29] and maturation timing in aquaculture Atlantic salmon.

Variation in the maturation phenotype explained by the resulting set of significant SNPs was estimated using the Monte Carlo average information restricted maximum likelihood method for variance component analysis implemented in BOLT-REML [39]. The model was defined as follows [4]:

$${\varvec{\upgamma}} = {\varvec{\upsigma}}_{0} \varvec{u}_{0} + {\varvec{\upsigma}}_{1} \varvec{Z}_{1} \varvec{u}_{1} + {\varvec{\upsigma}}_{2} \varvec{Z}_{2} \varvec{u}_{2} ,$$

where ${\varvec{\upgamma}}$ is a vector of phenotypes (0/1 for non-grilse/grilse), ${\varvec{\upsigma}}_{1} \varvec{Z}_{1} \varvec{u}_{1}$ and ${\varvec{\upsigma}}_{2} \varvec{Z}_{2} \varvec{u}_{2}$ are variance components to be estimated, and ${\varvec{\upsigma}}_{0} \varvec{u}_{0}$ is a random residual error effect. We partitioned the SNPs into two sets based on whether they were significant or not. Any SNP in high LD (*r*^2^ > 0.8) with a significant SNP was excluded from both sets. We then calculated the variance component for each set of SNPs. The variance in phenotype explained by each variance component was then estimated.

Heritability ($h^{2}$) of male maturation timing was inferred using the restricted maximum likelihood (–*reml*) method implemented in GCTA, which uses SNP-based relatedness estimates to calculate the proportion of phenotypic variance explained by a set of genotyped SNPs. The model was defined as follows:

$$h^{2} =\upsigma_{\text{u}}^{2} /\left( {\upsigma_{\text{u}}^{2} +\upsigma_{\text{e}}^{2} } \right),$$

where $\upsigma_{\text{u}}^{2}$ is the additive genetic variance explained by SNPs and $\upsigma_{\text{e}}^{2}$ is the error. We estimate $h^{2}$ with only the 50K SNP-array dataset because heritability estimates can vary with imputation certainty [46]. GCTA estimates $h^{2}$ on the observed (quantitative) scale, which we then transform to the underlying liability scale assuming varying levels of early maturation population prevalence (0.01, 0.025, 0.05) [47]. Due to the overrepresentation of the early maturation phenotype in this study sample, prevalence values below the sample prevalence (0.19) were used for transforming $h^{2}$ estimates from the observed scale to the liability scale.

## Results

### Genome-wide association testing

**Fig. 1:**
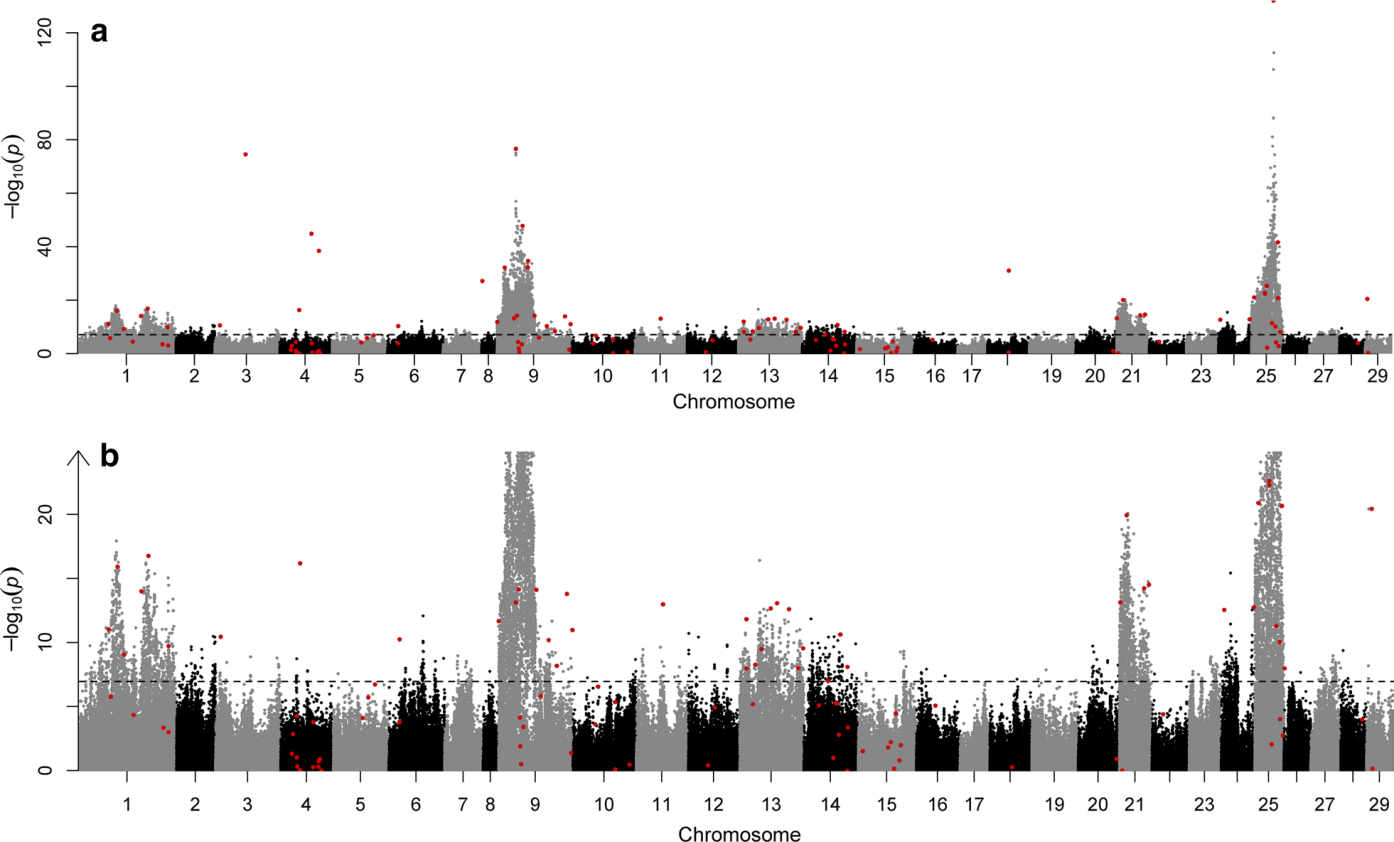
Manhattan plots for genome-wide association analysis of male early maturation. **a** Manhattan plot showing all SNPs. **b** Zoomed view of SNPs with association statistics below a –log_10_(*P*-value) of 25 (truncated Y-axis). The significance threshold (dashed line) was adjusted to account for multiple-testing using Bonferroni correction. Red dots indicate loci that were significant after conditional and joint analysis

Data on 11,166 males from the 2015 year-class imputed to 512,397 SNPs were used for association testing. The mean discordance between masked and actual genotypes for each chromosome ranged from 0.21 to 0.24 (see Additional file 1: Table S1). The linear mixed model association method showed that significant associations with maturation timing occurred on 28 of the 29 Atlantic salmon chromosomes (Fig. 1). In total, 13,149 of the 512,397 tested SNPs showed a significant association with maturation timing. A quantile–quantile plot of BOLT-LMM *P*-values indicated genomic inflation that is characteristic of a polygenic architecture of the trait [41] (see Additional file 2: Figure S1).

### Identification of candidate genes

**Fig. 2:**
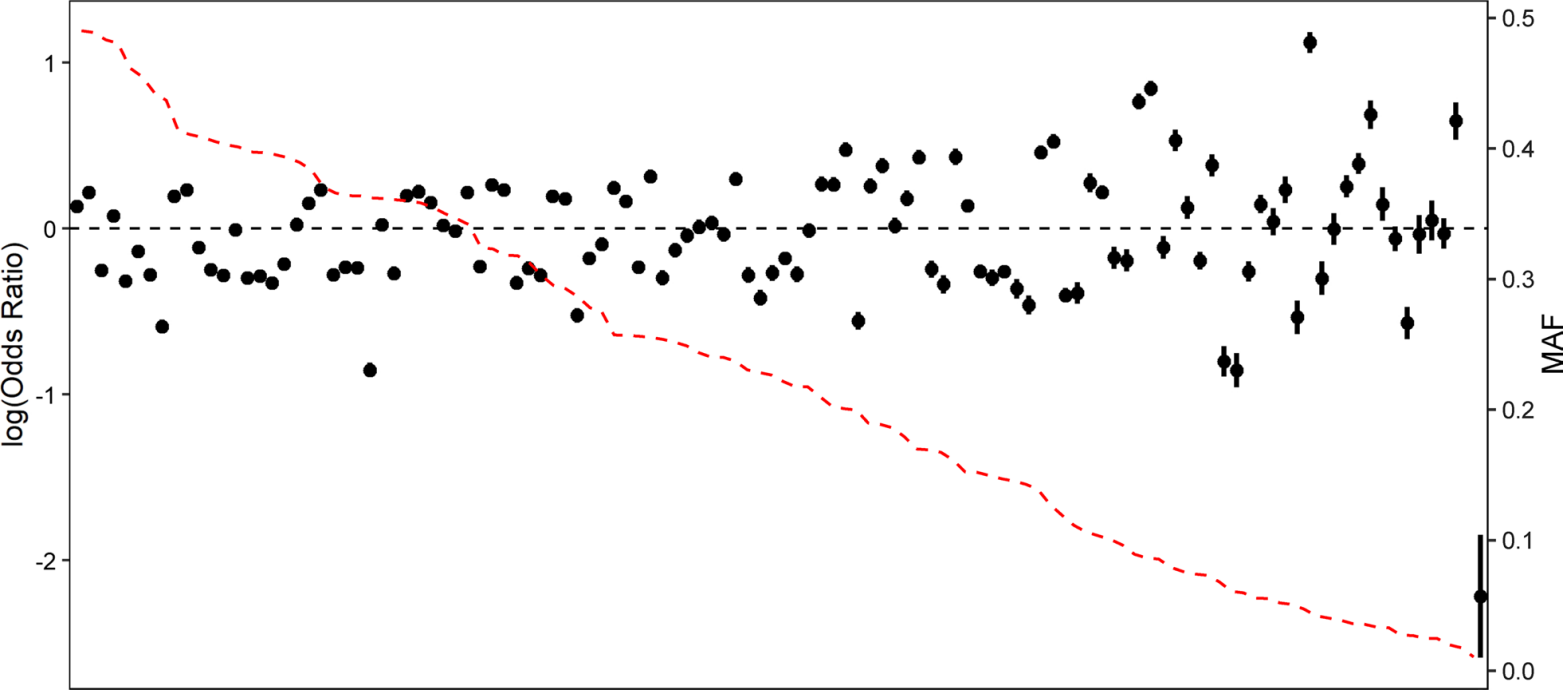
Minor allele frequency (MAF) (red line) and estimates of SNP effects on maturation relative to the major allele (black dots) as log-odds ratios, for the set of 116 independently associated SNPs (listed in Additional file 1: Table S2), ordered from largest to smallest MAF

**Fig. 3:**
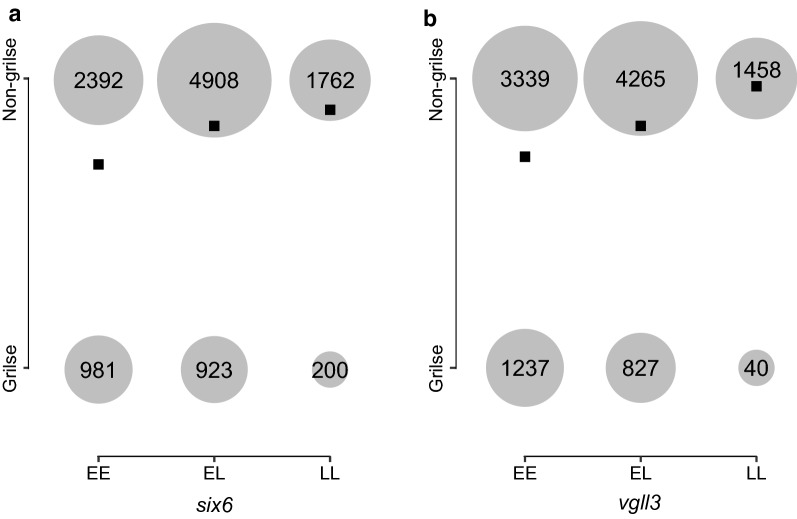
Number of grilse and non-grilse individuals with each genotype (*EE*, *EL*, *LL*) for **a** SNP tagging *vgll3* and **b** SNP tagging *six6*. Circles are proportional to sample size. *E* represents the allele that increases the odds of early maturation (early allele) and *L* represents the allele that decreases the odds of early maturation (late allele). Black squares indicate the mean phenotype value for each genotype (grilse = 1 and non-grilse = 2)

Conditional and joint analysis identified 116 SNPs that were independently associated with maturation time and reached genome-wide significance (Fig. 1) and (see Additional file 1: Table S2). These 116 SNPs were located on 22 of the 29 Atlantic salmon chromosomes. All of the 116 SNPs were on the 50K SNP array and, thus were not imputed. By selecting the gene in closest proximity to a significant SNP, this set of 116 SNPs tagged 120 candidate genes (see Additional file 1: Table S2). A SNP 251,183 bp downstream of a previously identified candidate gene, *vgll3* [1], was selected via conditional and joint analysis. *Vgll3* was not the closest gene to this SNP and, thus we assigned both the closest gene and *vgll3* as candidate genes tagged by this SNP. *Vgll3* was the only candidate gene assigned based on prior knowledge. For all other SNPs, the closest gene was assigned. For SNPs with a minor allele effect that increased the odds of early maturation, the odds ratios (OR) ranged from 1.01 to 3.07 (0- to 3-fold). The OR ranged from 0.11 to 0.99 (0- to 9-fold) for SNPs with a minor allele effect that delayed maturation (Fig. 2) and (see Additional file 2: Table S3). Five genes that were previously identified as candidate genes for age at maturity in humans were also candidate genes in this study. The first gene, *six6*, on chromosome 9, is tagged by an upstream SNP (9:24886574, OR = 0.55) (Fig. 3a). The second gene, *ndufs4*, is located on chromosome 15 and its tag SNP (15:6399839, OR = 0.74) is a missense variant. An intron SNP on chromosome 16 (16:27617999, OR = 1.70) tags the third gene, *rora.* Another intronic SNP on chromosome 22 (22:13016434, OR = 1.31) tags the fourth gene, *cntn4*. The fifth gene, *vgll3*, on chromosome 25 is tagged by a downstream SNP (25:28910202, OR = 0.42) (Fig. 3b) (see Additional file 1: Table S3).

Variance component partitioning was used to determine the proportion of variance explained by the set of 116 independently associated SNPs for maturation timing in male aquaculture salmon. The 116 SNPs explained 78% of the genetic variance, with the remaining 22% of the genetic variance explained by the remaining 512,244 SNPs in the imputed dataset.

GCTA estimates of the SNP-based $h^{2}$ was 0.61. After transforming to the liability scale, $h^{2}$ estimates ranged from 0.54 to 0.84, depending on underlying prevalence (0.01–0.05) (see Additional file 1: Table S4). Given these estimates, we can infer that 42.1, 53.8, and 65.5% of the phenotypic variance was explained by the set of 116 SNPs, for population prevalences of 0.01, 0.025 and 0.05, respectively.

## Discussion

Our sample size was substantially larger than in previous studies on the genetic basis of maturation of Atlantic salmon and focused on a single aquaculture strain, thus improving power and minimizing confounding effects of population stratification. This approach enabled us to look beyond large-effect loci and allowed us to reveal the polygenic component of maturation in Atlantic salmon. We confirmed the importance of a large effect locus in the *vgll3* region on chromosome 25 that was identified in previous studies [1, 15], but also identified several moderate effect loci (1.75- to 2-fold) including the *six6* locus on chromosome 9. The remaining loci identified either had smaller effects or they had large to moderate effects but low MAF (< 0.05). Furthermore, the number of additional regions of the genome that were associated with maturation suggests that this trait has a more polygenic architecture than previously indicated. The 120 candidate genes identified here provide a valuable resource for furthering our understanding of maturation in both aquaculture strains and wild populations. This work broadens the scope of empirical examples for the genetic architecture of quantitative traits, which is valuable for developing analytical frameworks to understand the genetic architecture underpinning quantitative traits in nature.

We observed a “new” highly significant SNP on chromosome 9, with an OR of 0.55, which remained significantly associated with age at maturity after correction for relatedness. This region was also identified in Barson et al. [1], but its association signal was lost after correction for population stratification in that study. Here, the most highly significant SNP in the region occurred ~ 16,000 bp upstream of *six6*, which has been increasingly recognized as a candidate gene for maturation in mammals [28, 31]. Population stratification correction is recommended to avoid spurious associations due to, e.g., systematic differences in ancestry between groups with different phenotypes [48], but there is the danger that it eliminates signals when trait-associated loci have different effects across populations (e.g. loci involved in local adaptation) [49]. Indeed, the *six6* region has been identified as potentially involved in local adaptation in numerous population genetic studies of Atlantic salmon. For example, the *six6* region is under divergent selection among Atlantic salmon populations in North America [50, 51] and the Teno/Tana River [30]. In addition, the *six6* gene has been associated with variation in run timing [30, 52] and river catchment area [30], and there is evidence that it plays a role in local adaptation in other fish species [53]. The potential role of *six6* in local adaptation may explain why its association signal was lost after population stratification correction in Barson et al. [1]. This highlights the importance of examining genotype–phenotype associations within single populations, in addition to multi-population studies. Together, these findings suggest that the role of the *six6* gene in determining age at maturity may vary among populations, but it should not be ruled out as a candidate gene for maturation in Atlantic salmon.

In this study, we also found that *vgll3* is important for controlling maturation, which agrees with some previous studies [1, 15], but not all [17, 19]. This may reflect continental differences in genetic architecture, with the locus-effect occurring only in the European lineage, or it may be due to low polymorphism in this region among North American derived aquaculture populations. Although we find a strong association of the *vgll3* region with maturation, which parallels that found in wild European salmon, we are not sure whether the same dominance pattern at the *vgll3* gene as observed in Barson et al. [1] exists here. Due to a trade-off between size and age at maturity, sexual conflict occurs in the wild, whereby late maturing alleles are favoured in females and early maturing alleles are favoured in males. In the *vgll3* gene, this conflict is resolved via sex-dependent dominance [1]. Based on proportions of late-maturing and early-maturing individuals for each genotype observed here, it does not appear that strong dominance of the early allele is at play in this aquaculture population (Fig. 3b). Genetic dominance is commonly defined as a deviation from a linear relationship between genotype dosage and effect on phenotype. However, here, sexual maturity is a binary trait and therefore does not follow a linear dose–response relationship. Thus, inferring dominance is complicated by this non-linear genotype dosage effect for binary traits. The pattern observed here, however, does suggest that sex-dependent dominance at this locus has been lost in this aquaculture strain. Indeed, factors suggested to cause sexual conflict, such as sex-specific maturation age and size optima, are eliminated in the aquaculture environment, since males and females are strictly selected for the same age at maturation. This suggests that genetic architecture can be rapidly altered in a setting with weak or no sexual conflict, which may be plausible, e.g. via modified expression and/or methylation patterns [54, 55]. Changes to dominance patterns in response to environmental changes have been previously described in some organisms [56, 57].

In addition to the *six6* and *vgll3* genes, we identified 114 other candidate loci with varying effect sizes, demonstrating a mixed genetic architecture that underlies maturation in Atlantic salmon (i.e., a small number of large-effect genes combined with a polygenic component). These 114 additional candidate loci included nine loci with moderate to large effects (1.75 < fold) across six chromosomes (4, 8, 9, 10, 24 and 29), and numerous smaller effect loci (1.75 > fold) distributed across 21 chromosomes (see Additional file 1: Table S2). These findings provide evidence that maturation in Atlantic salmon is a polygenic trait and reveal many new candidate genes that underlie variation in timing of maturation. In addition, we found some overlap (5 genes) between the candidate genes identified here and candidate genes for age at maturity in humans [28, 29]. This suggests that some aspects of the genetic control of the timing of maturation may be conserved across evolutionarily distant species.

Interestingly, a number of SNPs found to be associated with maturation in this study were low-frequency variants with moderate (1.75- to 2-fold) to large (two- to ninefold) effect sizes (Fig. 2). Such low-frequency variants have been a topic of interest in human genetics research [58, 59]. The “rare allele model” has been proposed as the reason for the missing heritability issue [60]. It suggests that low-frequency alleles with large effects can contribute to a particular phenotype at the population level, whereby rare alleles at a particular locus explain most of the variation in just a small number of individuals, but when such rare, large effect alleles occur at many loci, the collective contribution of rare alleles can be large at the population level. However, it has also been shown that low MAF can cause an inflated number of false positives in GWAS and biases in effect sizes [61–63]. Therefore, further validation of these large-effect low-frequency variants is recommended.

Candidate genes were identified via conditional and joint analysis and, therefore, represent independent signals, i.e., they are not merely the result of being in LD with another associated locus. Although the candidate genes assigned based on proximity to these signals are plausible, we cannot conclude that these are the causal genes for differences in age at maturity. Instead, the causal gene may be further upstream or downstream. Future annotation of non-coding variation in the Atlantic salmon genome will help to validate SNP-to-gene assignment. Furthermore, although some candidate loci had small independent effects, they were still identified as candidates because their association with age at maturity was highly significant when considered in combination with other loci (see Additional file 1: Table S2). This situation can arise when a genotyped SNP does not account for the total amount of variation explained by a QTL (e.g. [64, 65]), or when multiple causal variants exist at a single QTL (e.g. [2, 66]). Based on the selection criteria used here, we consider these loci as strong candidates in spite of their small independent effect size.

The maturation-associated QTL identified here provide a valuable contribution to our understanding of how aquaculture strains can respond to selection. Multiple smaller effect QTL indicate that there is an opportunity for fine-tuning of the trait via approaches such as genomic selection—an opportunity that would not exist if the trait was controlled by a single large-effect locus. Furthermore, this work suggests that a polygenic approach to selective breeding aimed at optimizing maturation timing may be beneficial. Focusing on just a few QTL (e.g. *six6* and *vgll3*) may not be sufficient to prevent early maturation, as there are a number of other QTL that may modify maturation timing. In addition, considerations of this mixed genetic architecture are valuable for designing effective management and conservation strategies of wild Atlantic salmon. Maintenance of variation in age at maturity is of ecological, economic, and cultural importance [67]. Factors such as fishing [21] and ecological changes in the marine environment [68] could affect early- and late-maturing individuals differently, which could lead to reduced variation in sea age [69]. Our improved understanding of the genetic architecture for maturation can help to more accurately predict the effects of such factors. This study also exemplifies how smaller effect genes can be missed until a sufficiently high-powered analysis is used. For this reason, even when large-effect loci are identified, it is important that management and conservation strategies consider remaining genetic variation and continuously aim at maintaining genome-wide variation [70].

We did not perform association testing for loci involved in female maturation due to the very low occurrence of female grilsing in this strain. From an aquaculture perspective, this low prevalence also implies that determining the genetic basis of maturation in females is of lower importance in farmed strains such as this one. However in the wild, early maturation in females is more commonly observed and, therefore, studies aimed at refining the genetic architecture of this trait in females would benefit future research on wild populations. In addition, because we focused on only one strain, determining the generality of the mixed architecture of maturation identified here requires assessment of additional wild populations and aquaculture strains.

## Conclusions

We refined our understanding of the genetic architecture of maturation of male Atlantic salmon using a large-scale GWAS. We revealed a polygenic component of age at maturity in Atlantic salmon and identified several moderate- and large-effect loci. The 120 candidate genes identified here can serve as a valuable resource for furthering our understanding of maturation in both aquaculture strains and wild populations. These results also help to elucidate how this trait will respond to factors such as fishing and environmental changes in the wild.

## Supplementary information

**Additional file 1: Table S1.** Mean discordance between imputed and actual genotypes per chromosome. **Table S2.** List of the 120 candidate genes tagged by the 116 significant SNPs identified with conditional and joint analysis. The *P*-value indicates significance of the association test performed in BOLT-LMM using a non-infinitesimal model. P-value (COJO) indicates the significance of the SNP after conditional and joint analysis. Beta and its corresponding standard error (Beta_SE) were estimated using BOLT-LMM and indicate the effect size of the minor allele. The odds ratio (OR) and its corresponding transformed standard error (SE_T) were calculated using LMOR. **Table S3.** List of all 512,397 SNPs in the final dataset. Output statistics from BOLT-LMM (p-value, Beta & Beta_SE) and LMOR (OR, log(OR) & SE_T) are listed. The p-value indicates significance of the association test performed in BOLT-LMM using a non-infinitesimal model. Beta and its corresponding standard error (Beta_SE) were estimated using BOLT-LMM and indicate the effect size of the minor allele. The odds ratio (OR) and its corresponding transformed standard error (SE_T) were calculated using LMOR. **Table S4.** Estimated heritabilities from GCTA using the 50K array dataset. Estimates are given on the observed quantitative scale and when transformed on the liability scale assuming population prevalences of 1%, 2.5% and 5%.

**Additional file 2: Figure S1.** Quantile–quantile plot of association *P*-values from BOLT-LMM showing residual inflation following linkage disequilibrium score regression calibration expected under polygenicity.

## Data Availability

The data that support the findings of this study are the property of AquaGen AS and restrictions apply to their availability, which were used under license for the current study. Thus, the data are not deposited on a public repository, but can be accessed under agreement with AquaGen AS.
