# Supplementary material for: Beyond large-effect loci: large-scale GWAS reveals a mixed large-effect and polygenic architecture for age at maturity of Atlantic salmon
Source: Genet Sel Evol. 2020 Feb 12;52:9. doi: 10.1186/s12711-020-0529-8 (PMC7017552; doi:10.1186/s12711-020-0529-8)
Supplement: Supplementary file 2 — Additional file 2: Figure S1. Quantile–quantile plot of association P-values from BOLT-LMM showing residual inflation following linkage disequilibrium score regression calibration expected under polygenicity. [file 12711_2020_529_MOESM2_ESM.docx]

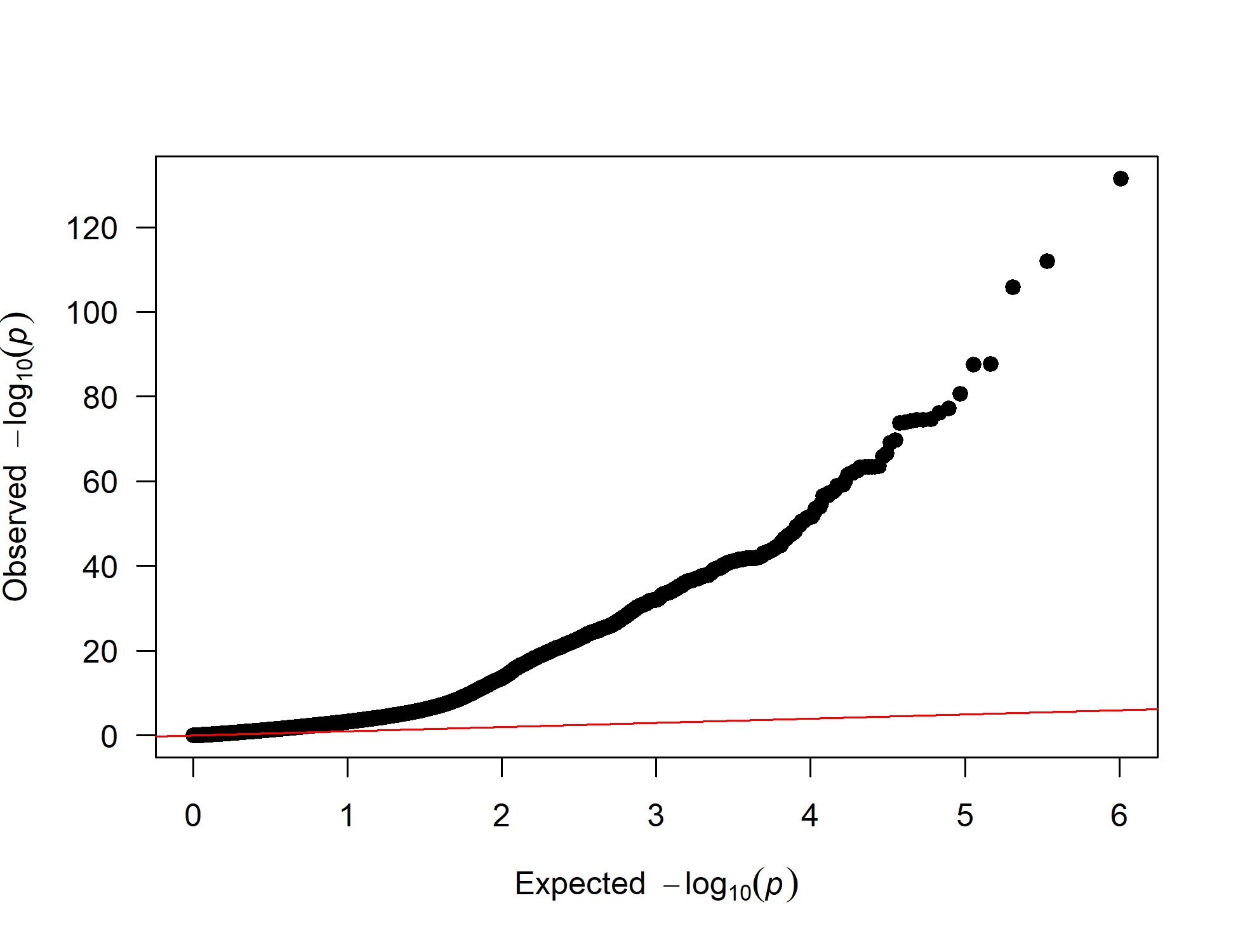


Figure S1. Quantile-quantile plot of association *P-*values from BOLT-LMM showing residual inflation following linkage disequilibrium score regression calibration expected under polygenicity.
